# The effect of COVID vaccination timing on the seroprevalence of IgG antibodies: evidence from the Guayas region of Ecuador

**DOI:** 10.3389/fpubh.2025.1537049

**Published:** 2025-03-25

**Authors:** Aurora Malacatus-Arboleda, Erick Barbotó-Ramírez, Gonzalo E. Sánchez, Bernard Moscoso, Lauren A. Rhodes, Josefina Coloma, Ángel Guevara, Fernando Espinoza-Fuentes, Juan Carlos Fernández-Cadena, Gabriel Morey-León, Derly Andrade-Molina

**Affiliations:** ^1^Laboratorio de Ciencias Ómicas, Facultad de Ciencias de la Salud, Universidad Espíritu Santo, Samborondón, Ecuador; ^2^Facultad de Ciencias Sociales y Humanísticas, Centro de Investigaciones Económicas, Escuela Superior Politécnica del Litoral, ESPOL, Guayaquil, Ecuador; ^3^Facultad de Ciencias Sociales y Humanísticas, Centro de Investigaciones Rurales, Escuela Superior Politécnica del Litoral, ESPOL, Guayaquil, Ecuador; ^4^Division of Infectious Diseases and Vaccinology, School of Public Health, University of California, Berkeley, Berkeley, CA, United States; ^5^Instituto de Biomedicina, Carrera de Medicina, Universidad Central, Quito, Ecuador; ^6^Harvard Medical School and Brigham and Women’s Hospital, Boston, MA, United States

**Keywords:** COVID-19, SARS-CoV-2 RBD, IgG, cross-sectional study, vaccine, *in-house* ELISA assay, seroprevalence

## Abstract

**Background and aims:**

Timely distribution of COVID-19 vaccines was particularly important for developing countries that do not have strong health systems and related infrastructure. We analyze data from the Guayas province of Ecuador, an area particularly affected by the pandemic, to determine the seroprevalence of SARS-CoV-2 and the effect of the timing of the second dose of COVID-19 vaccines on the seroprevalence SARS-CoV-2 IgG antibodies.

**Methods:**

This cross-sectional study involved 1,761 individuals aged 18 and older who voluntarily enrolled prior to and during the initial phase of vaccine rollout in Ecuador (October 2020 to July 2022). IgG anti-SARS-CoV-2 RBD antibodies were assessed by an *in-house* ELISA to evaluate the immune response to Pfizer (BioNTech, Spike mRNA) and AstraZeneca (Oxford, AstraZeneca Spike) vaccine in the Guayas province. Ordinary least squares (OLS) regressions were employed to determine the effect of delayed second doses later than prescribed by the manufacturer for both vaccines.

**Results:**

Before the vaccination campaign, we estimated an RBD IgG seroprevalence of 27.7% (95% CI: 23.6–27, *n* = 469). The estimate increased to 89.4% (95% CI: 87.7–91.18, *n* = 1,235) after the first vaccine dose and to 92.6% (95% CI: 90.7–94.5, *n* = 748) after the second dose. Individuals who received the second dose of the Pfizer vaccine later than the recommended dose showed significantly lower levels of IgG antibodies 2–3 weeks after receiving the second dose than those who received the dose within the recommended timeframe. Furthermore, we did not find any effect on RBD IgG antibody levels in those who received a second dose of the AstraZeneca vaccine during the first and second parts of the recommended vaccination window.

**Conclusion:**

The results suggest that a significant portion of the study population was already infected with SARS-CoV-2 prior to the vaccination. As expected, seropositivity increased alongside vaccination efforts. We determined that Pfizer vaccine recipients should be adhered to vaccine timing guidelines. Furthermore, resource-limited countries should consider administering vaccines with flexibility in dosing intervals, such as AstraZeneca, as it allows for a wider time frame without significantly reducing the boosting of IgG antibodies.

## 1 Introduction

In late 2019, in Wuhan, China, a new pneumonia-causing SARS-CoV-2 coronavirus was identified, which caused the Covid-19 pandemic (1, 2). This virus left the world in a virtual standstill until the first vaccine was widely distributed in December 2020. The presence of COVID-19 vaccines was particularly important for developing countries, where the lack of infrastructure and the organization of their public health systems left health centers vulnerable to collapse from the massive surge of infections during the initial months of 2020. Guayaquil, Ecuador, was one of the hardest cities in Latin America, gaining notoriety for its massive mortality during the initial COVID-19 wave (3, 4). The Ecuadorian government launched a national vaccination plan in January 2021, which was deemed exemplary globally owing to its rapid implementation, resulting in a decline in the number of deaths and severe cases (3). Initially, the vaccination strategy prioritized healthcare workers and the older adult with the Pfizer vaccine, which was the first available vaccine purchased by the government. The AstraZeneca vaccine was later administered to the general population in addition to the Chinese vaccine Sinovac. The original vaccine schedule for both Pfizer and AstraZeneca between 2020 and 2022 required the administration of two doses, with the aim of increasing protection against SARS-CoV-2, thereby reducing virus transmission and minimizing hospital admission (5, 6).

Vaccine makers and the WHO Strategic Advisory Group of Experts on Immunization (SAGE) provided a wide window in which to receive a second dose (such as AstraZeneca), while others provided a tighter recommended timeframe (such as the Pfizer vaccine). While guidelines on when to receive second doses and boosters were recommended, not everyone chose to follow these guidelines, and there was variance in when the second doses and boosters were received in Ecuador. The panorama of COVID-19 vaccination evolved in early 2023 with the introduction of bivalent vaccines, which are updated annually to address emerging variants, such as Omicron (7). These bivalent formulations, authorized as booster doses, provide enhanced protection against severe outcomes. However, in Ecuador, bivalent vaccine doses only arrived in July 2023 and have mainly been applied to vulnerable groups. This campaign ended in September 2023 by applying approximately 262,000 doses (8).

The administration of the second dose of the COVID vaccine produces an increase in humoral and cell-mediated immune responses (9, 10), resulting in broader protection against SARS-CoV-2 compared to patients who received a single dose of a vaccine without a previous COVID infection (9). Nevertheless, in the first years of vaccination, some governmental agencies around the world have explored the advantages and potential drawbacks of extending the time intervals between the second doses and booster shots beyond the officially recommended schedules. Although numerous studies have concentrated on investigating the consequences of vaccination delays, the safety implications of these actions remain uncertain (11–14). In Ecuador, this issue remains incompletely elucidated, particularly regarding its impact on immunogenic responses.

IgG antibodies represent the predominant type of antibodies within the bloodstream, which exhibit the longest serum half-life among all immunoglobulins. The Receptor Binding Domain (RBD) and spike proteins are employed to detect these antibodies in SARS-CoV-2 positive patients, as SARS-CoV antibodies target the RBD epitopes, providing information on the future identification of antibodies with cross-reactivity and greater neutralizing activity (15, 16). Therefore, tests for anti-SARS-CoV-2-RBD IgG are the gold standard for determining seroprevalence. Consequently, anti-SARS-CoV-2 IgG antibodies serve as markers for identifying past infections, including asymptomatic or mildly symptomatic cases, as well as for assessing the level of immunogenic response elicited by vaccination (17). Some studies have shown that when measuring the seroprevalence of spike IgG of SARS-CoV-2 in individuals, the level of this antibody is higher in the tripled-vaccinated population than in those who received a primary vaccine scheme/standard vaccine regimen of two doses (18). However, the immune response triggered by the vaccine strongly depends on host characteristics (age, history of past Covid-19 infections, genetics, comorbidities, and gender) and vaccine-related factors (vaccine types, number of doses, and standard vaccine regimen). Nonetheless, receiving a regular booster has demonstrated an increase in humoral responses in the general population compared with the standard initial dosing regimens (19).

Most adults in Guayaquil (Guayas) (approximately 85.26%) received at least one dose of a COVID-19 vaccine during our study period (20). This high proportion of vaccinated individuals provides an opportunity to observe how the timing of second doses and boosters affected the seroprevalence of SARS-CoV-2 RBD IgG for different COVID-19 vaccines, highlighting the importance of evaluating seroprevalence levels of SARS-CoV-2 IgG antibodies before and after the vaccination process and through the correlation between the vaccination regimen and IgG antibody production using the highly sensitive and accessible in-house ELISA method in developing countries such as Ecuador. Understanding these dynamics is essential, as variations in the interval between the first and second doses have been shown to influence antibody responses over time.

Several studies have suggested that extending the interval between vaccine doses can enhance antibody responses by allowing for a more robust maturation of the immune system before the booster dose (14, 21). However, this effect may vary depending on the vaccine platform, population characteristics, and prior exposure to SARS-CoV-2. Still, the optimal dosing schedule remains a topic of debate, particularly in regions where vaccine availability and logistics influence administration timing.

In this study, we use the data from SARS-CoV-2 IgG antibodies in the Guayas province of Ecuador to evaluate IgG seroprevalence before and after vaccination and correlate it with different vaccination regimens. The main contribution of this paper is to analyze the effect of the timing of the second dose of COVID-19 vaccines on the seroprevalence SARS-CoV-2 IgG antibodies.

## 2 Materials and methods

### 2.1 Study design and participants

This study is a cross-sectional analysis that assessed anti-SARS-CoV-2 RBD IgG positivity using an *in-house* ELISA assay and vaccination history of Guayas province residents over 18 years of age who voluntarily enrolled between late 2020 to mid-2022. Our cohort includes 1,761 participants with 2,667 tests, divided by age groups in two sub-studies. The first study evaluated the antibody seroprevalence before the first vaccination. The second study evaluated the IgG dynamics of the participants who received only the first and second doses. The age ranges for the analysis were 18–23, 24–29, 30–39, 40–49, 50–59, 60–69, and above 70 years old (22). This study was approved by the Expedited Ethics Committee of the Ecuadorian Health Ministry (MSP-024-2020). All biological and epidemiological data from the patients were anonymized.

### 2.2 Sample processing and IgG detection by ELISA

Human serum samples were obtained with informed consent from all participants. The collected samples were handled and transported in strict compliance with the guidelines established by the World Health Organization. Serum specimens were preserved at −30°C for later use in ELISA assays (23).

To determine the seroprevalence of COVID-19, the indirect RBD ELISA assay was employed with some modifications to the protocol originally developed by Stadlbauer et al (24). Recombinant protein RBD was donated by the Icahn School of Medicine at Mount Sinai Philanthropic Organization, through Aubree Gordon, University of Michigan; which was authorized for emergency use by the U.S. Food and Drug Administration (FDA) (25). Briefly, flat-bottom 96-well plates (NuncMaxisorp™, Thermo Fisher Scientific, Waltham, MA) were coated with 50 μL of a 2.5 μg mL recombinant protein RBD, resuspended in 1X PBS, pH 7.2 (Gibco, Invitrogen) and incubated overnight at room temperature. The solution was removed, and 100 μL of blocking buffer (4% BSA, 0.1% Tween 20, 1X PBS Buffer) was added and incubated for 30 min at room temperature. Following the incubation period, the wells were washed five times with the wash buffer PBST (1X PBST, 0.1% Tween 20). Serum samples from patients as well as positive and negative controls were diluted 1:100 in PBST with 5% skim milk (dilution buffer) and incubated at 37°C for 1 hour. The plates were washed five times with 270 μL washing buffer. Then, 100 μL of goat anti-IgG human horseradish peroxidase (Invitrogen, 31,410, Rockford, IL, USA) diluted 1:8000 in dilution buffer was added to each well and incubated at 37°C for 30 min. The wells were washed seven times with 270 μL PBST. Finally, 100 μL of o-phenylenediamine dihydrochloride and fresh substrate solution (Thermo Fisher Scientific) were added to each well and incubated in the dark for 10 min. The reaction was stopped by adding 100 μL of 3 N HCl. Absorbance was measured at 490 nm using a Synergy HTX (BioTek/Multi-Mode Microplate Reader Synergy HTX). The cut-off value was calculated as the mean ± 3 standard deviations (SDs) of the absorbance values from pre–COVID-19 sera. Samples with an absorbance equal to or greater than the cut-off value were considered positive (15).

### 2.3 Statistical data processing and analysis

The *in-house* ELISA validation titles were driven using RStudio obtaining a 99% confidence interval (CI) for the estimated statistics. Receiver-operating characteristic (ROC) analysis was performed on optical density (OD) ELISA data to determine the area under the curve (AUC) and optimal cutoff point (26–28). Samples were categorized as either positive or negative according to the cut-off determined by ROC analysis for each test. The sensitivity and specificity of the ELISA test data were determined by calculating the area under the curve (AUC), using the standardized OD values obtained from the ELISA tests. We obtained the Youden index to evaluate the efficacy of the ELISA in the demographic cohort of this study (29). Conversely, the statistical concordance between the gold standard previous diagnostic method RT-qPCR using Allplex™ 2019-nCoV Assay (Seegene Inc., Seoul, Republic of Korea) and the ELISA test was evaluated through Cohen’s kappa coefficient. Throughout all our analyses, the threshold for considering a *p*-value statistically significant was <0.05.

Subsequently, all data were analyzed using the Stata Statistical Software Release 16 (Stata Corp LLC, College Station, TX, Stata Corp LLC). We began with a descriptive statistical analysis of the sample. Regression analysis was used to study the relationship between the timing of the second vaccine dose and the seroprevalence of IgG antibodies. Specifically, we use ordinary least squares regression with the following basic structure:


$$IgG_{i}=\beta_{0}+\beta_{1}X_{1i}+u_{i}$$

*IgG* is the level of antibodies present 2–3 weeks after receiving the second dose of either Pfizer or AstraZeneca vaccine. *X_1i_* is an indicator variable that takes the value of one if the second dose was administered 29 days or more after the first dose for Pfizer, and between 36 and 84 days after the first dose for AstraZeneca vaccine. We extended this basic specification and add control variables for sex (a binary indicator that equals 1 if individual is a female and equals 0 if individual is a male), city of residence (a binary indicator that equals 1 if the individual resides in the city of Guayaquil, and 0 otherwise), age of the patient, and month of the year’s fixed effects. The standard errors are robust to heteroskedasticity.

## 3 Results

Initially, our study evaluated the performance of the ELISA test in our diverse population. The efficacy and diagnostic capability of the ELISA assay for the identification of IgG antibodies targeting the RBD were systematically assessed through ROC analysis. ROC analysis demonstrated a strong diagnostic capability, with an AUC of 0.85 (CI 0.7394–0.9662) and a low standard error (0.0579), indicating a precise estimate. The test showed high sensitivity (100%) and specificity (98%) (Supplementary Figure 1). Additionally, the Kappa coefficient (0.83, CI 0.73–0.95) suggested ‘almost perfect agreement’ with RT-PCR, and the Youden index supported an optimized cut-off value of 0.98. These results demonstrate the high reliability, accuracy, and consistency of the ELISA test in identifying both positive and negative cases (Supplementary Table 1).

Following this performance assessment, we proceeded with the analysis of the study samples, which included a total of *N* = 2,667 RBD ELISA tests from a total of individuals of *N* = 1,761. Out of the total ELISA tests, *N* = 1,094 are tests from males and *N* = 1,573 are from females. Approximately 13% of all samples come from those between the ages of 18 and 23, 15% from those between the ages of 24 and 29, 26% from those between the ages of 30 and 39, 22% from those between the ages of 40 and 49, 15% between the ages of 50 and 59, 7% between the ages of 60 and 69, and 3% come from those who were at least 70 years old. Table 1 provides the seroprevalence of SARS-CoV-2 antibodies by gender, city of residency, and age group prior to vaccination.

**Table 1:** Age-stratified seroprevalence of SARS-CoV-2 RBD antibodies and gender and age groups among ELISA tests from individuals with at least 1 vaccine.

Variable	Samples collected	Crude prevalence	95% CI	Mean difference	*p-*value
Gender					
Total	1,235	0.8947	(0.8775972–0.9118765)	–	–
Male	477	0.8910	(0.8629–0.9191)	–	–
Female	758	0.8971	(0.8754–0.9188)	0.006	≥0.1
Age group (years)					
18–23	167	0.8683	(0.8164–0.9201)	–	–
24–29	208	0.9519	(0.9226–0.9812)	0.084	≤0.01
30–39	325	0.8800	(0.8445–0.9155)	0.012	≥0.1
40–49	257	0.8833	(0.8437–0.9228)	0.015	≥0.1
50–59	171	0.9064	(0.8623–0.9505)	0.038	≥0.1
60–69	77	0.8701	(0.7933–0.9469)	0.002	≥0.1
>70	30	0.9000	(0.7861–1.0139)	0.032	≥0.1
Residency					
Guayaquil	950	0.8947	(0.8752–0.9143)	–	–
Other	285	0.8947	(0.8589–0.9306)	0.000	≤0.05

Prior to the start of the vaccination campaign, the overall seroprevalence among all participants was found to be 27.7% (95% CI: 23.6 to 27.7%). Seroprevalence in younger individuals in the age group of 18–23, those aged 60–69 and people above 70 years showed higher seroprevalence rates, at 36.8% (95% CI: 20.7 to 52.9%), 38.8% (95% CI: 22.1 to 55.6%), and 41.6% (95% CI: 8.9 to 74.3%), respectively. This contrasts with the RBD seroprevalence observed in the economically active population age group of 24–59 years, who had 25.3% (95% CI: 20.9 to 29.7%). It is important to note that there were no significant differences in seroprevalence levels between the male and female participants (Table 2).

**Table 2:** Age-stratifies seroprevalence of SARS-CoV-2 RBD antibodies and gender groups among ELISA tests from individuals before vaccination.

Variable	Samples collected	Crude prevalence	95% CI	Mean difference	*p*-value
Gender					
Total	469	0.2772	(0.2365273–0.2771855)	–	
Male	232	0.2802	(0.2219–0.3383)	–	–
Female	237	0.2744	(0.2170–0.3314)	−0.006	≥0.1
Age group (years)					
18–23	38	0.3684	(0.2077–0.5291)	–	–
24–29	71	0.2535	(0.1498–0.3572)	−0.115	≥0.01
30–39	137	0.2262	(0.1553–0.2972)	−0.142	≤0.1
40–49	107	0.2710	(0.1854–0.3566)	−0.097	≥0.1
50–59	68	0.2794	(0.1699–0.3888)	−0.089	≥0.1
60–69	36	0.3888	(0.2216–0.5561)	0.020	≥0.1
>70	12	0.4166	(0.0895–0.7438)	0.048	≥0.1
Residency					
Guayaquil	387	0.2662	(0.2219–0.3104)	–	–
Other	82	0.3293	(0.2254–0.4332)	0.063	≤0.1

Table 1 shows the seroprevalence by primary vaccination status, excluding those who had never received a COVID-19 vaccine. In this table we observe an increase in the overall seroprevalence of 89.4% following the initial dose, in contrast to the data of previous vaccination presented (Table 2), reflecting an increase of 61.7%. When only considering those who had received at least one vaccine, there is no significant difference between males and females.

**Figure 1:**
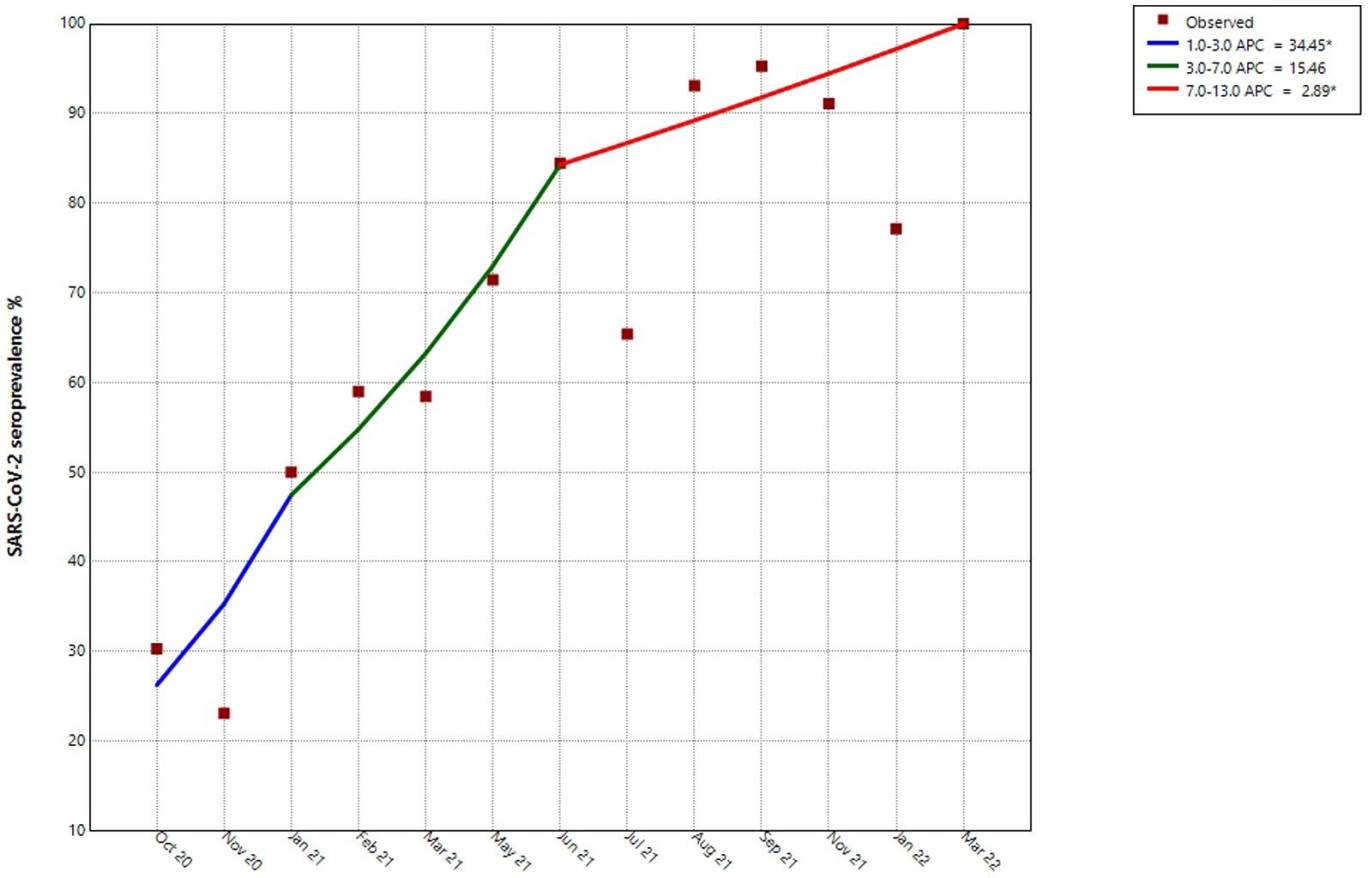
Joinpoint analysis of the levels of seroprevalence of SARS-CoV-2 over time. This figure shows the months in which samples were collected during the ELISA testing program. *indicates that the percentage change is significantly different from zero at the alpha = 0.05 level.

The seroprevalence of SARS-CoV-2 RBD antibodies was measured in the sample population from October 2020 to March 2022. The joinpoint regression analysis indicates three different trends (Figure 1), a steady but small increase before the vaccination period. Whereas higher seropositivity trend was observed at the time of the Ecuadorian vaccination campaign in February of 2021. However, in subsequent months, a steady rise in seroprevalence within the population was observed, reaching 92% by September 2021 due to the third vaccine dose along with the presence of the Delta variant and 100% by March 2022. This 100% seroprevalence was achieved thanks to pre-existing immunity from vaccines and the presence of the Omicron variant, which led to high peaks of infection.

**Figure 2:**
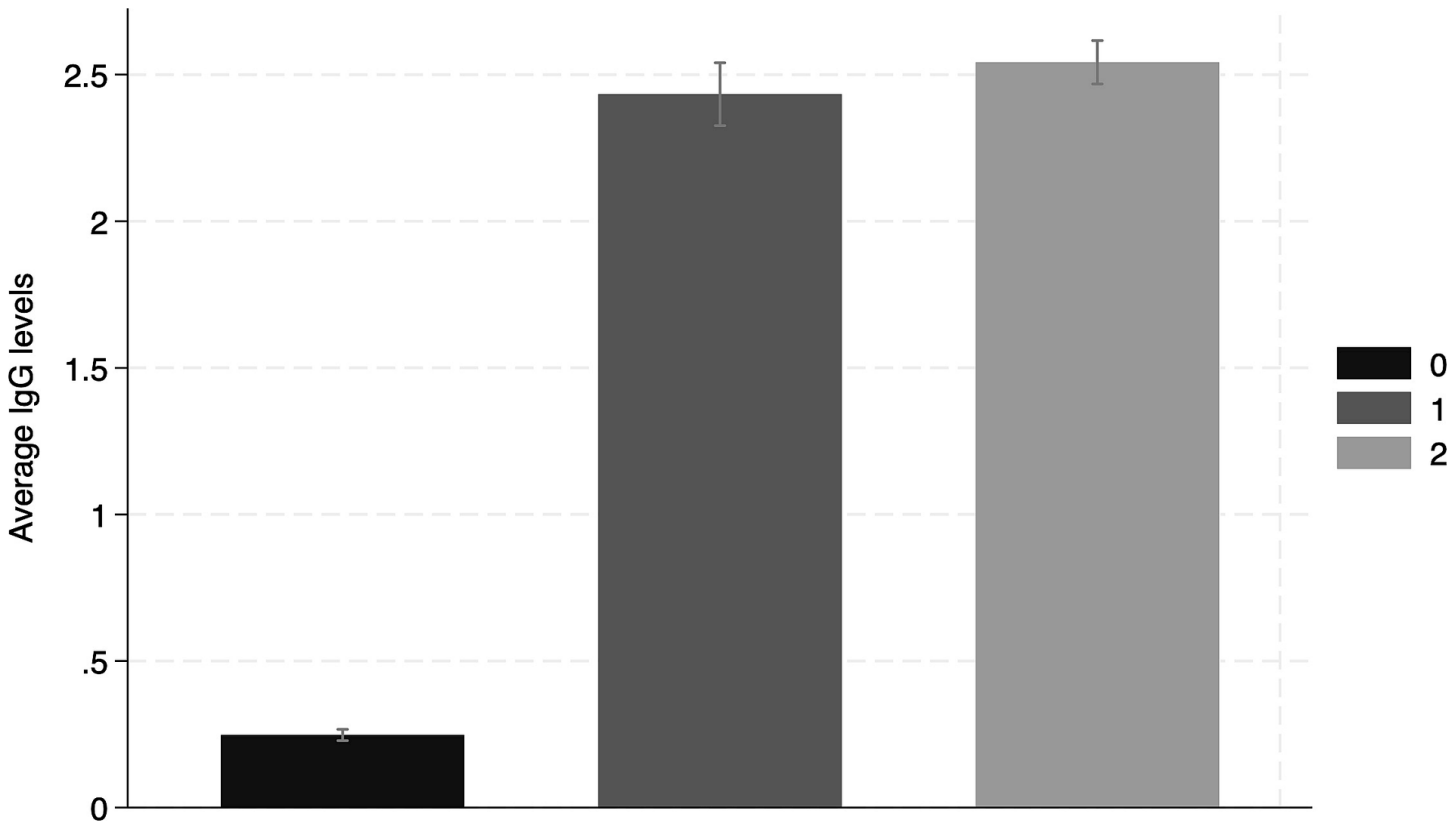
Average seroprevalence of IgG RBD antibodies according to the number of vaccines received.

While it is promising to see an upward trend of seroprevalence, the previous figure is not enough alone to demonstrate how RBD IgG antibodies are influenced by vaccine doses. Figure 2 shows the average RBD IgG levels by the number of received vaccines. For those who had not received vaccines, we only considered those who had never tested positive for COVID up to the point of observation. We observe that there is a significant difference in the level of RBD IgG between those who did not receive any vaccines and those who received at least one vaccine. For the purposes of this study, we did not analyze the level of RBD IgG after the third dose of the vaccine, as it was a highly biased group that had received the third vaccine at the time of this study. As the Ecuadorian government phased risk groups to receive vaccines (the older adult, frontline workers, and the immunocompromised received vaccines first), there were likely characteristic differences between those who had the opportunity to receive a third dose during the time of this program (the immunocompromised, for example) and those who had not yet had the opportunity to be administered a third dose.

The timing of the second dose of the vaccine also appears to have played an important role in the average RBD IgG levels, depending on the vaccine administered. We looked at the Pfizer and AstraZeneca recombinant vaccines and only considered those who had received their second vaccine dose within 2–3 weeks of the ELISA test. This was to ensure that sufficient antibody levels were boosted in the individuals, and that we had a comparable group of observations.

**Figure 3:**
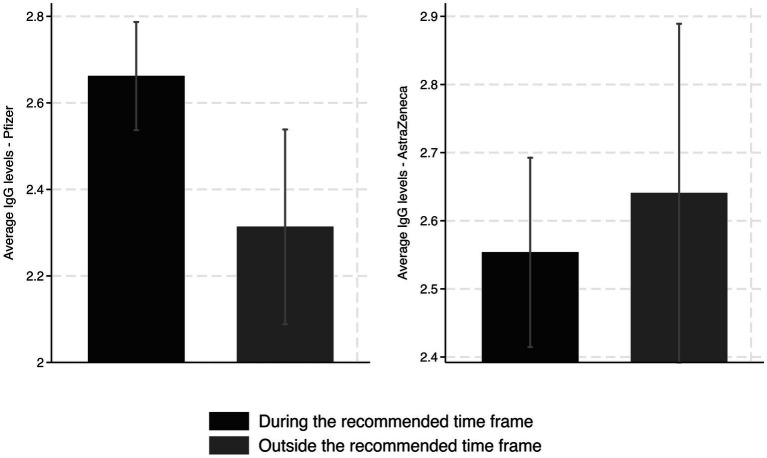
Average RBD IgG levels from those who received the second dose of the Pfizer (left) or AstraZeneca (right) vaccines, during or outside of the recommended time frames after the first dose, respectively (95% C.I).

Our analysis of the Pfizer vaccine only considered those who had received Pfizer vaccines for both their first and second doses. For these mRNA vaccines, the second dose was recommended to be administered between 21 and 28 days after receiving the first dose. A second dose of 81.59% (*n* = 297) received a second dose between 21 and 28 days, whereas 18.41% (*n* = 67) received the second dose 29 days or more after the first dose. As shown in Figure 3, there appeared to be a significantly lower average RBD IgG level when the vaccine was administered late.

Our regression analysis confirms that this lower level of IgG is around 0.36 (OD_450_ nm), which is robust across different specifications. Column 4 of Table 3 shows the most specifications accounting for gender, city, age, month of the year, and fixed effects. The estimated coefficient on the variable *29 days or more* shows a decrease of about 0.39 (OD_450_nm) [95% CI: −0.7671 to −0.0093; *p* = 0.0446]. These findings are equivalent to a reduction of approximately 14.5% concerning those who received the second dose in the recommended time frame, implying the importance of adhering to vaccination schedules as outlined by the manufacturer.

**Table 3:** Estimates of the effect of delayed second Pfizer vaccine dose on average RBD IgG levels.

	IgG levels			
	(1)	(2)	(3)	(4)
29 days or more	−0.3484^**^	−0.3830^***^	−0.3215^**^	−0.3882^**^
	(0.1437)	(0.1453)	(0.1615)	(0.1925)
	[0.0159]	[0.0088]	[0.0474]	[0.0446]
Constant	2.6619^***^	–	–	–
	(0.0668)			
	[0.0000]			
Female		X	X	X
Guayaquil		X	X	X
Age			X	X
Month of the year fixed effects	–	–	–	X
Observations	364	364	364	364
*R* ^2^	0.0158	0.0204	0.0241	0.3996

Robust standard errors are indicated in parentheses. *p*-values in square brackets. Female and Guayaquil are binary variables representing observations coming from a female and person living in Guayaquil, respectively. Significance levels: **p < 0.05, ***p < 0.01.

It was recommended that those who received the first dose of AstraZeneca vaccine wait between 28 and 84 days to receive the second dose. Due to this large time frame, the vast majority of people received their second dose within this recommended window. Only three people in our sample received their second vaccine early (one received the second vaccine 25 days after the first vaccine, and two received the second vaccine 27 days after the first vaccine). Therefore, we observed differences in IgG levels within the early part of this timeframe (28–35 days) and the later part of the timeframe (36–84 days). Of the observations, 74.78% (*n* = 169) were within the 28-to-35-day time frame and 25.22% (*n* = 57) were within the 36-to-84-day time frame. Figure 3 shows the average RBD IgG levels separated between these two groups among those who received AstraZeneca for both their first and second doses. While the average RBD IgG levels were higher for those who waited longer in our sample, it appears that the difference is not significant. Our regression analysis (Table 4) confirmed that there was no significant difference in RBD IgG levels based on whether the vaccine was applied in the earlier or later part of the acceptable window.

**Table 4:** Estimates of the effect of receiving the second AstraZeneca vaccine dose between 28 and 35 days after the first dose on average IgG levels.

	IgG levels			
	(1)	(2)	(3)	(4)
35 days or less	−0.0870	−0.0764	−0.0993	−0.1646
	(0.1450)	(0.1437)	(0.1471)	(0.1502)
	[0.5490]	[0.5957]	[0.5001]	[0.2747]
Constant	2.6406^***^	–	–	–
	(0.1249)			
	[0.0000]			
Female		X	X	X
Guayaquil		X	X	X
Age			X	X
Month of the year fixed effects	–	–	–	X
Observations	226	226	226	226
*R* ^2^	0.0017	0.0182	0.0231	0.1545

Robust standard errors are indicated in parentheses. *p*-values in square brackets. Female and Guayaquil are binary variables representing observations coming from a female and person living in Guayaquil, respectively. Significance levels: ***p < 0.01.

## 4 Discussion

The distribution of COVID-19 vaccines is vital for the functioning of the health system in Ecuador and other developing countries, and for a return to normalized societal activities (30, 31). Epidemiological surveillance estimated through the seroprevalence of SARS-CoV-2 plays a critical role in understanding the extension of infections in the population, estimating immunological protection, and evaluating the effectiveness of vaccines across different demographic groups. This information is important for public health strategies and the development of national policies aimed at responding effectively to pandemics (32, 33). The seroprevalence data obtained through ELISA has provided insights into the spread and risk factors associated with infections. Testing specificity and sensitivity in each study with in-house ELISA tests is crucial to ensure the accuracy and reliability of the diagnostic tool.

In our study, to ensure that our results are reliable, the ELISA test was evaluated by ROC curves that demonstrated high performance in distinguishing between positive and negative cases, showed an AUC of 0.85, with high sensitivity (100%) and specificity (98%), which is consistent with previous findings of IgG seropositivity studies performed in Ecuador with a sensibility of 93.6% and specificity of 100% (34) and suggests a reliable application across diverse populations (35). The high PPV of 0.971 and NPV of 1 suggest that these tests effectively minimize both false positives and false negatives, making them suitable and trustworthy for diagnostic use. Overall, these metrics highlight the reliability and robustness of both ELISA and the general diagnostic model for an accurate diagnosis. The substantial agreement (kappa of 0.83) observed in the ELISA test further supports its consistency in comparison with a reference standard, reinforcing its suitability in both clinical and epidemiological settings. However, despite their high performance, an AUC below 1 suggests room for further refinement to achieve an even higher accuracy in differentiating cases. Ensuring diagnostic reliability is crucial for accurately interpreting seroprevalence trends, as it guarantees that fluctuations in antibody presence reflect genuine epidemiological changes rather than methodological inconsistencies.

In our cohort from Guayas, we observed a pre-vaccination seroprevalence of 27.7%, suggesting significant early SARS-CoV-2 exposure in the study population. However, this rate may differ from higher seroprevalence reported in specific Ecuadorian populations, such as pregnant women and neonatal cord blood samples (34), as well as rural coastal regions, where seroprevalence reached 43% (35). These differences could be attributed to several factors, including variations in population density, socioeconomic conditions, healthcare access, and exposure risks. This findings underscores the relevance of conducting seroprevalence studies within local communities and considering regional and demographic variations when analyzing seroprevalence to assess the actual impact of the pandemic across different populations to generate tailored public health strategies.

Accurately capturing temporal variations in seroprevalence requires reliable serological testing methods capable of distinguishing between antibodies from natural infections and vaccine-induced immunity. It is essential to employ techniques that provide precise measurements while distinguishing between these two types of immunity. Various serological techniques include chemiluminescent immunoassays, immunofluorescence assays, and rapid immunochromatographic tests that can quantify anti-RBD IgG antibodies (36–38); they are not the sole marker of infection. However, these techniques help to discriminate between natural infection with anti-NP IgG antibodies and immunity following vaccination with anti-RBD IgG antibodies (39). Due to their high cost, these methods are not a cost-effective option for large population studies, particularly in resource-limited settings such as Ecuador. In contrast, ELISA offers an affordable alternative for seroprevalence studies with high sensitivity and specificity while remaining accessible for large-scale implementation.

Considering the limitations of expensive serological techniques, it is essential to also evaluate the broader public health measures, particularly the role of vaccination in controlling the spread of SARS-CoV-2. Vaccination was crucial in increasing protection against SARS-CoV-2, reducing virus transmission, and minimizing hospital admissions. This was particularly evident during the emergence of the Omicron variant, which led to the highest number of recorded positive cases in Ecuador throughout the pandemic. However, despite this surge in infections, a significant increase in seroprevalence was observed, highlighting the combined impact of natural infection and widespread vaccination efforts. In addition cellular immunity is essential for long-term protection. Pfizer and AstraZeneca vaccines trigger strong IgG responses. Both vaccines generate memory T cells that remain effective against variants. Furthermore, combining vaccines may enhance overall protection and reinforce the importance of diverse immunization strategies (40).

In our study, immediately after the administration of the initial vaccine dose against SARS-CoV-2, the seroprevalence surged to 89.4% on average, irregardless of the time of the administration, exhibiting a 61.7% increase in RBD IgG seropositivity in our study population. After the second dose, irregardless of the administration date, the seroprevalence was 92.6% on average. These findings in our study are similar to the global seroprevalence trends, underscore the relevance of our research in the pandemic context, and highlight the importance of the vaccination process. In view of this, the tracking of seroprevalence is vital not only for determining whether the vaccines have been effective but also for monitoring the progression and spread of the virus, planning new public health policies, updating the existing knowledge about seroprevalence, and how it differs in specific populations globally (41, 42).

Our general results align with those of similar studies in Japan, where the same antigen was used with a sensitivity of 92.5% and a specificity of 100% (25). It also aligns with the results from developing countries where the pandemic generated a major impact on the population, such as the Central African Republic, where the seroprevalence study showed 31.8% before vaccination and 97.5% after vaccination (43), Chile presented 22.3% pre-vaccination and 88% post-vaccination (44), and India reported 31.5% seroprevalence before vaccination and increased to 93.1% after completing the vaccination schedule (45). In contrast, a study conducted in Canada reported higher seroprevalence of anti-RBD IgG antibodies in individuals who received their second dose of Pfizer after 89 days or more (46). This discrepancy may be attributed from differences in statistical models, antibody quantification methods and baseline immunity, as our cohort had a 27.7% pre-vaccination seroprevalence, which could have influenced immune responses. Additionally, the other study may have included longer delays (6–8 weeks), linked to stronger antibody responses.

Similarly, studies with results comparable to ours found that, based on the categorization of our population, most of who participated in our ELISA project were the population between 24 and 59 years of age who presented a seroprevalence of 25.5% (CI: 20.9–29.70%). We show that the estimated seroprevalence among the subsets of participants in the Labour force between 25 to 64 years had lower seroprevalence than other age groups. This aligns with previous reports, where the workforce was more vigilant about avoiding infection and often underwent diagnostic tests more frequently to seek to sustain their economic activities (47).

Our findings also suggest the importance of receiving a second dose of the COVID-19 vaccine within the period recommended by the manufacturer and WHO SAGE. According to our results, not receiving the second dose resulted in a lower average ELISA positivity, interpreted as lower IgG antibodies depending on the type and administration of the vaccine. People who received the second dose of Pfizer vaccine after the recommended time window had a lower level of IgG antibodies than those who received the dose at the time indicated by the manufacturer, while for people who received the second dose of AstraZeneca within the recommended administration window, IgG levels remained steady. Therefore, it is essential that healthcare providers and government health organizations promote and provide second doses in the correct timeframe.

Although not delaying the second dose is important, we found that seroprevalence was statistically unaffected by the administration of the second dose within the recommended time frame. This was examined for AstraZeneca, which has a generous window for receiving a second dose. Therefore, countries that may have infrastructural limitations for administering doses should a new wave of the coronavirus pandemic occur considered to promote the AstraZeneca vaccine, as it allows for more variance in the administration of the second dose. The administration of the initial two doses of COVID vaccines during the window period provided increased protection by reducing infection, hospitalization, and mortality rates (48–50). This is because a minimum of two doses of the vaccine are required during the timeframe to achieve the best vaccine efficacy (50). In our study, it was demonstrated that adherence to the timeframe for the Pfizer vaccine resulted in higher values in patients after the first two vaccines compared to those who received no doses. This aligns with similar studies in Saudi Arabia, Brazil, and Chile (51–54), indicating an enhanced immunological memory. In Chile, similar results were obtained for the AstraZeneca vaccine doses (55).

The vaccine programs for preventable diseases served as a basis for developing initial guidelines for the timing of coronavirus vaccination programs and establishing similarities and solutions for challenges during the SARS-CoV-2 Pandemic (56). Comparing the traditional timing immunization campaigns for diseases such as measles or polio, which have phased and structured protocols; COVID-19 vaccines were rapidly developed and distributed in response to the global pandemic emergency; this accelerated process led to administrative and logistical problems, affecting routine immunization programs worldwide (57). However, the pandemic prompted innovative strategies such as co-administration of anti-SARS-CoV-2 vaccines with influenza vaccines (58). Nonetheless, the global emergency revealed the importance of equity in vaccine distribution, especially for marginalized regions to access immunization timely. These approaches underscored the need for adaptable vaccination programs to contain future pandemics while maintaining accessibility and efficiency in response to evolving epidemiological trends (59).

At the same time, as vaccine development advances, it is crucial to assess not only efficacy in terms of immunogenicity but also the safety profiles of each platform. While mRNA vaccines have demonstrated superior immune responses, each technology presents unique characteristics that require continuous monitoring (60). The recent withdrawal of AstraZeneca in some regions because it has been linked to cases of thrombocytopenia and blood clots (61), highlights the importance of considering alternative platforms, to expand available options and ensure the best possible immune response in different contexts.

### 4.1 Study limitation

This study had some limitations. First, due to our limited sample size, as this study was conducted at only one testing and administration facility, we did not have a completely random sample of people across Ecuador and absence of information of prior infection data in most of the population. Therefore, our study cannot be generalized to the entire Ecuadorian population. Second, is the inability to distinguish between IgG seropositivity resulting from natural infection and that induced by vaccination. This is due to the lack of access to anti-NP IgG antibody testing, which is specific to natural SARS-CoV-2 infection. Consequently, our findings reflect overall IgG seroprevalence without differentiating the source of immunity. However, given the significance of our findings, the results of this study should be considered in future vaccine administration policies. Furthermore, our study opens the door for future replication studies to be conducted using different sample populations and vaccines. Third, more health or sociodemographic information could have been included to add more variables to the presented models. This is because we lacked access to the precise timing of the first-time infection among participants with previous infection. Fourth, it is possible that asymptomatic infections occurring after serological assessment of serostatus may restrict the interpretation of immune protection.

## Data Availability

The raw data supporting the conclusions of this article will be made available by the authors, without undue reservation.

## Supplementary material

SUPPLEMENTARY FIGURE 1 Evaluation of the efficacy of the ELISA tests through receiver-operating characteristic (ROC) analysis.
