# Supplementary figures and images for: The effect of COVID vaccination timing on the seroprevalence of IgG antibodies: evidence from the Guayas region of Ecuador
Source: Front Public Health. 2025 Mar 25;13:1537049. doi: 10.3389/fpubh.2025.1537049 (PMC11975873; doi:10.3389/fpubh.2025.1537049)

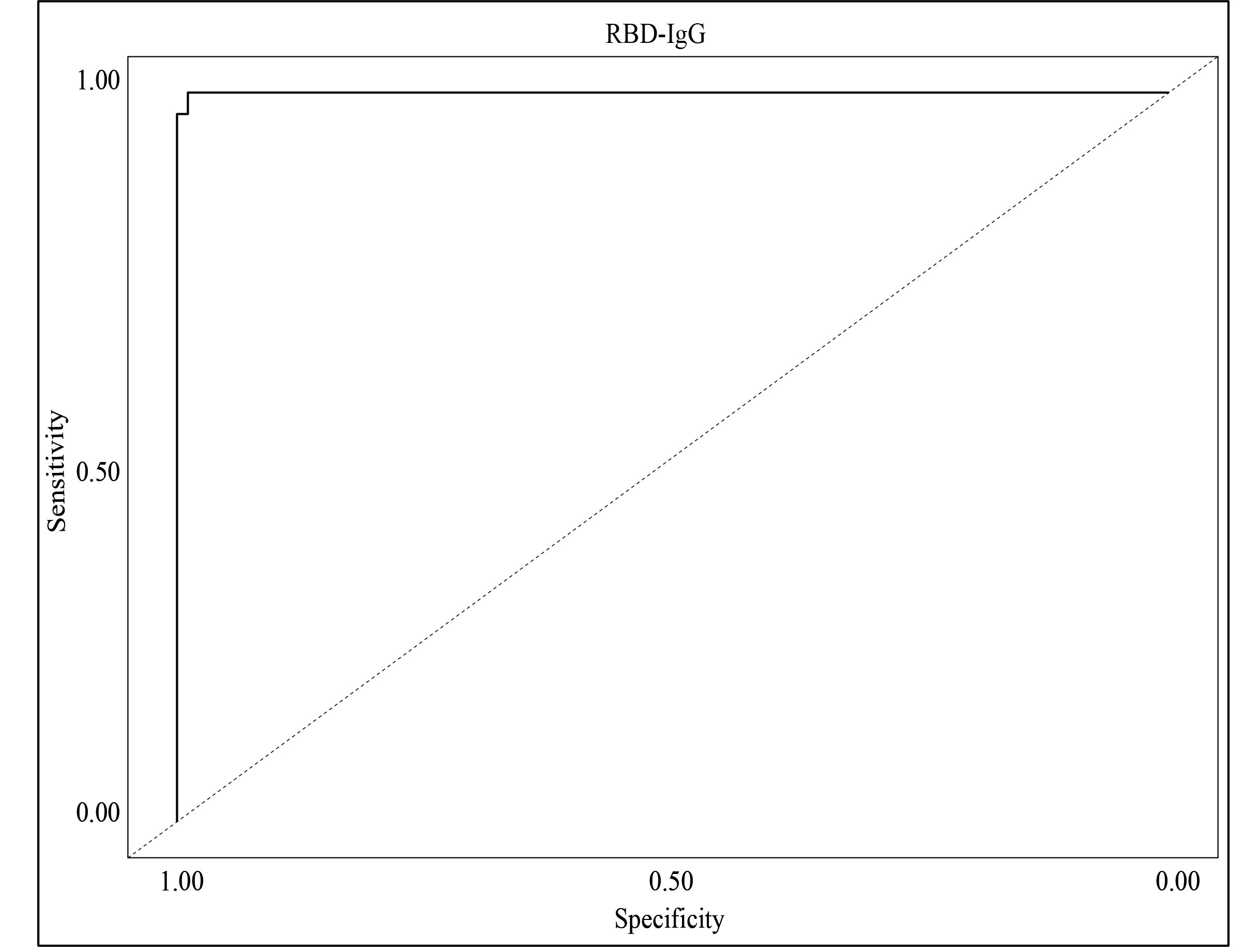

Supplement: SUPPLEMENTARY FIGURE 1 — Evaluation of the efficacy of the ELISA tests through receiver-operating characteristic (ROC) analysis. [file Image_1.JPEG]
